# Monocular eye position specificity in the oculomotor neural integrator

**DOI:** 10.1186/1471-2202-12-S1-P151

**Published:** 2011-07-18

**Authors:** Naoki Okamura, Robert Baker, Yutaka Hirata

**Affiliations:** 1 Dept. Computer Science, Chubu University Graduate School of Engineering, Kasugai, Aichi, 487-8501, Japan; 2 Dept. Physiology and Neuroscience, New York University School of Medicine, New York, NY, 10065, USA

## Background

The oculomotor neural integrator (NI) is a conceptual neuronal mechanism that maintains eye position after each scanning saccade without visual feedback in the dark. Saccades are triggered by an impulse-like activity of brain stem burst neurons that, similar to a mathematical function, must be integrated to produce a step-like motor command. The impulse- and step-like signals are combined in motor neurons to produce a rapid saccadic eye movement followed by a stable eye position. In the Laplace domain, a perfect integrator is described as 1/*s* and an imperfect one as 1/(*s*+*a*) with a positive *a* being “leaky” and a negative *a*, “unstable”. Experimentally, the NI was found to be modifiable by using visual feedback paradigms to imitate either ‘leaky’ or ‘unstable’ behaviors [1]. In goldfish, neurons exhibiting a highly correlated NI activity have been found in the hindbrain Area I [1]. A recent study demonstrated 3 types of neurons in Area I: ipsilateral (37%), conjugate (59%), and contralateral (4%) [2]. The ipsi- and contralateral neurons increased their firing rate when the ipsi- and contralateral eye positions changed in the temporal and nasal direction, respectively. By contrast, the conjugate neurons did not distinguish between the eyes and fired in both cases. This finding predicts that if the visual feedback paradigm would be applied exclusively to either a nasal or temporal hemi-field of one eye, the effect of training would not be confined to the trained hemi-field.

## Methods

To test this prediction, we conducted visual feedback NI training in goldfish. The fish were gently restrained at the center of a cylindrical water tank with a planetarium projecting random dots on a white wall. Eye position was monitored by the search coil technique while spontaneous scanning saccades occurred in both directions about a central neutral position. For monocular visual-feedback NI training, the L eye was occluded and the planetarium rotated by a servomotor at a speed proportional to R eye position. When planetarium motion was either centrifugal or centripetal to the selected neutral position, the NI could be trained to be either leaky or unstable, respectively. In the current study we employed R eye unstable only training of both hemi-fields or only one hemi-field (nasal or temporal) by turning off the planetarium when eye position was in the other hemi-field.

## Results

Nasal only unstable training (n=8) made the NI significantly unstable in the trained R eye hemi-field, but leaky in the untrained R eye hemi-field. The training also affected the NI for the untrained L eye such that it was leaky in the nasal and unstable in the temporal hemi-field. Combined nasal and temporal unstable training (n=9) resulted in a significantly unstable NI for the trained nasal hemi-field, but almost no change in the trained temporal hemi-field. In the untrained L eye, the NI was significantly unstable in the temporal hemi-field but almost no change in the nasal hemi-field. In contrast, temporal only unstable training (n=9) produced just a small change in the NI for that hemi-field and an even smaller leak in the untrained nasal hemi-field. In the L eye there was a small change towards unstable in the nasal hemi-field with hardly any difference in the temporal hemi-field.

## Conclusions

The present results demonstrate that unstable training of a single hemi-field makes the NI leaky for the opposite hemi-field of the same eye as well as ‘unstable and leaky’ for hemi-fields in the untrained eye. This experimental finding was predictable from ipsilateral and conjugate Area I neuronal activity [2]. However, the small amount of learning after temporal only and even less after both temporal and nasal unstable training was not foreseen. These results are being further evaluated directly by recording from Area I neurons throughout training and memory.

## References

[B1] Major G Baker R Aksay E Mensh B Seung HS Tank DW Plasticity and tuning by visual feedback of the stability of a neural integrator Proc Natl Acad Sci USA 2004 101 7739 7744 15136746 10.1073/pnas.0401970101 PMC419676

[B2] DeBowy O Baker R Encoding of eye position in the goldfish horizontal oculomotor neural integrator J. Neuropysiol 2011 105 2 896 909 10.1152/jn.00313.2010 PMC4103780 21160010

